# Supplementary material for: Prioritization of putatively detrimental variants in euploid miscarriages
Source: Sci Rep. 2022 Feb 7;12:1997. doi: 10.1038/s41598-022-05737-3 (PMC8821623; doi:10.1038/s41598-022-05737-3)
Supplement: Supplementary file 1 — Supplementary Figures. [file 41598_2022_5737_MOESM1_ESM.pdf]

Supplementary Figures

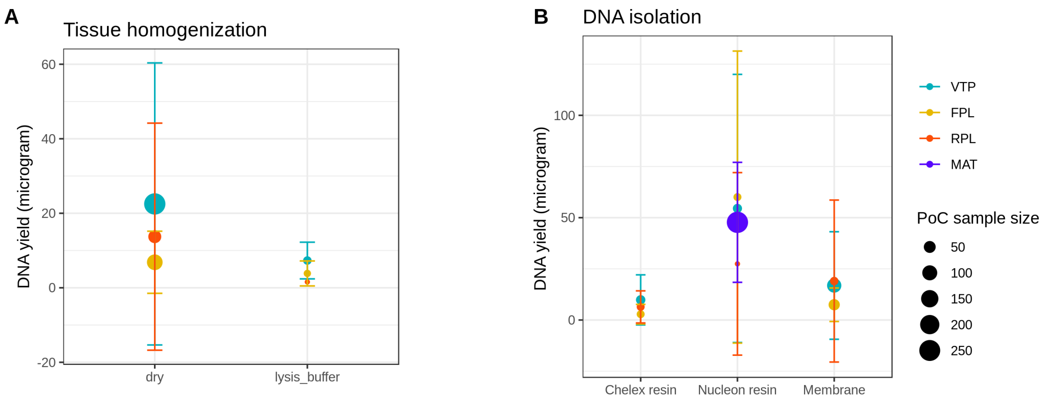

Figure S1. Optimization of tissue homogenization and DNA extraction. We do not observe significant difference between two methods of tissue homogenization (**A**), and three methods of DNA isolation (**B**) apart form a slightly higher range of yield for one type of resin. VTP: voluntary pregnancy termination; FPL: first pregnancy loss; RPL:recurrent pregnancy https://www.overleaf.com/project/60892471a11e466a8e7a0b18loss; MAT: maternal bllod; PoC: product of conception.

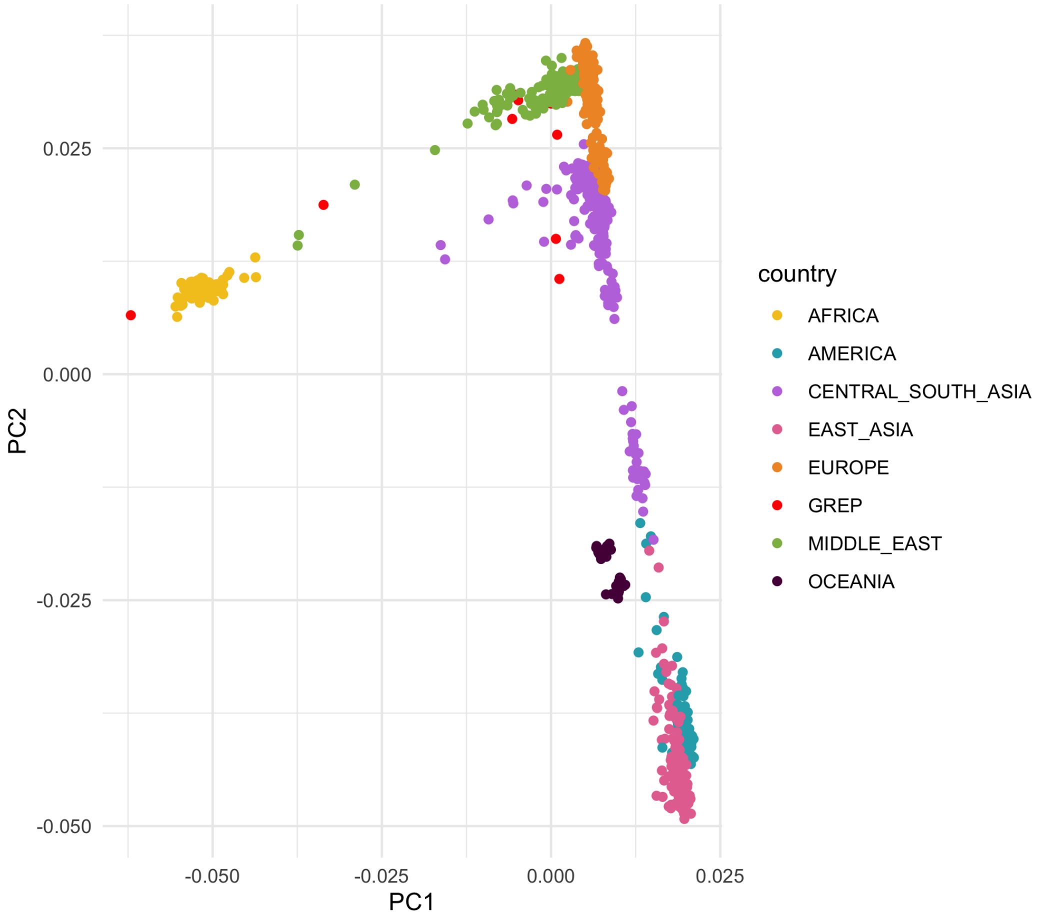

Figure S2. **Principal Component Analysis** of embryos from this study (GREP) and individuals in the HGDP panel used as controls
